# Supplementary material for: Structural and Morphological Studies of Pt in the As-Grown and Encapsulated States and Dependency on Film Thickness
Source: Nanomaterials (Basel). 2024 Apr 20;14(8):725. doi: 10.3390/nano14080725 (PMC11054750; doi:10.3390/nano14080725)

Supplementary Materials

Structural and Morphological Studies of Pt in the As-Grown and Encapsulated States and Dependency on Film Thickness

Figure S1. (**a–e**) and (**k–o**) are the HR-RSM data of the Pt111 and Pt222 reflections, respectively, for Pt_Th10nm, PtTh25nm, Pt_Th40nm, Pt_Th55nm and Pt_Th70nm in the as-grown state. **(e1, e2**) and (**o1, o2**) are the corresponding angular and radial diffraction profiles of the Pt111 and Pt222 reflections, respectively. **(f–j**) and (**p–t**) are the HRRSM of the Pt111 and Pt222 reflections, respectively, for EncPt_Th10nm, EncPtTh25nm, EncPt_Th40nm, EncPt_Th55nm, and EncPt_Th70nm in the encapsulated state. (**j1, j2**) and (**t1, t2**) are the corresponding angular and radial diffraction profiles of the Pt111 and Pt222 reflections, respectively.

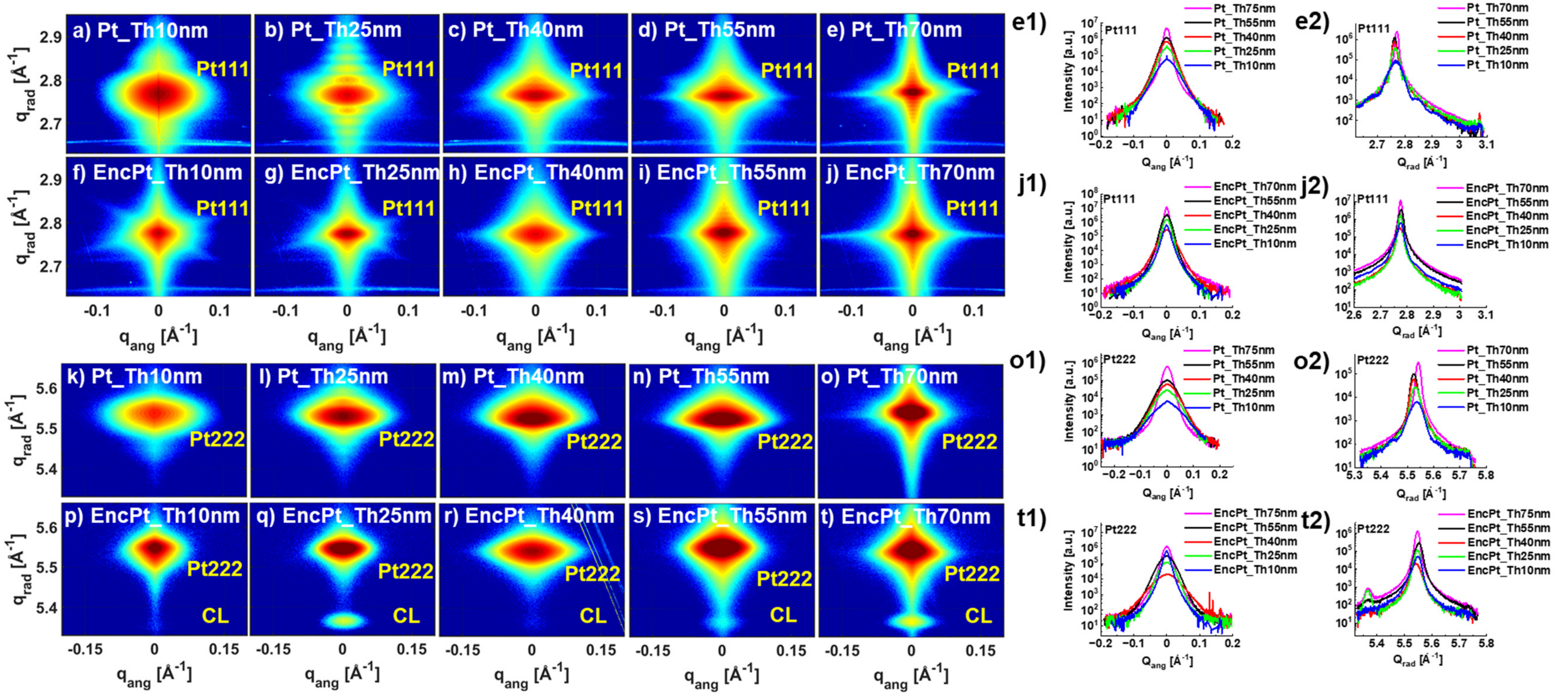

Figure S2. (**a–e**) and (**f- j**) are the combined HR-RSMs of Pt331 and Y224 asymmetric reflections of the Pt_Th10nm, Pt_Th25nm, Pt_Th40nm, Pt_Th55nm, and Pt_Th70nm, as well as the corresponding data of EncPt_Th10nm, EncPt_Th25nm, EncPt_Th40nm, EncPt_Th55nm, and EncPt_Th70nm, respectively.

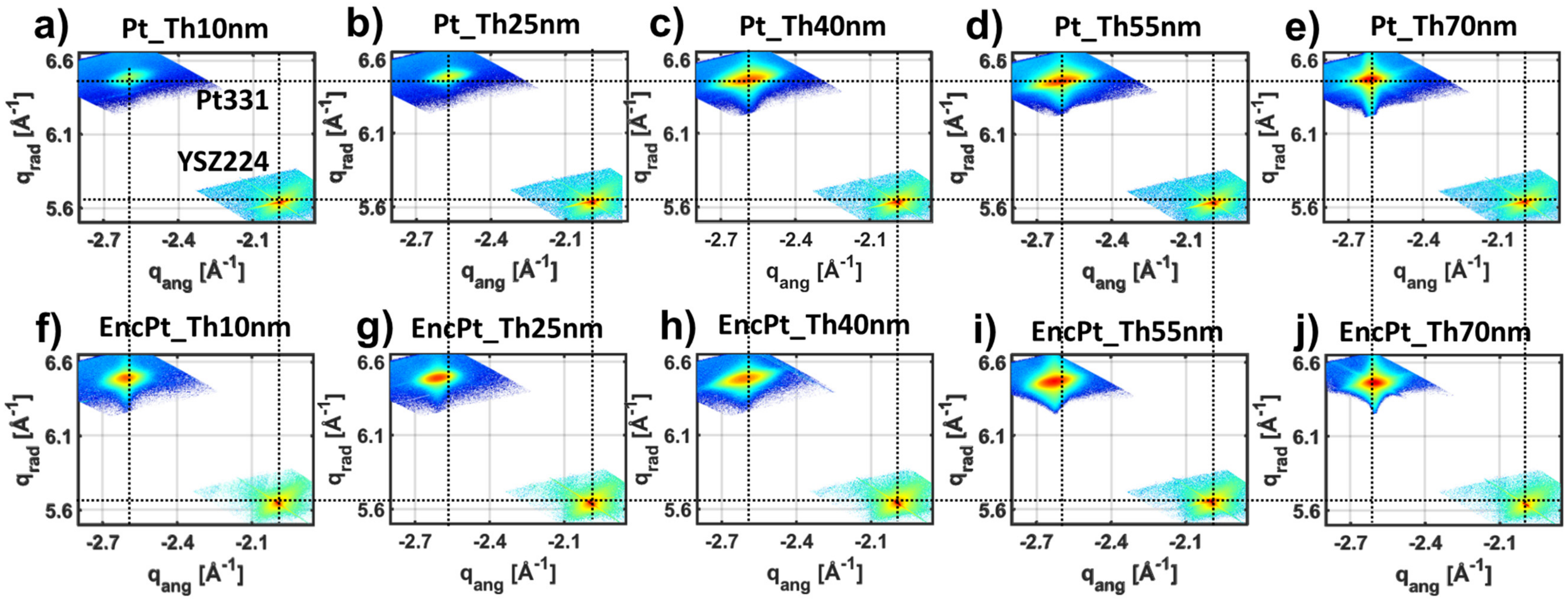

Figure S3: (**a–e**) are the BSE_0° images for the samples Pt_Th10nm, Pt_Th25nm, Pt_Th40nm, Pt_Th55nm, and Pt_Th70nm in the as-grown state. (**f–j**) BSE_0° images of the samples in the encapsulated state, i.e. EncPt_Th10nm, EncPt_Th25nm, EncPt_Th40nm, EncPt_Th55nm, and EncPt_Th70nm. The degree of coverage was derived from the images and is given in percentage in the BSE_0° images.

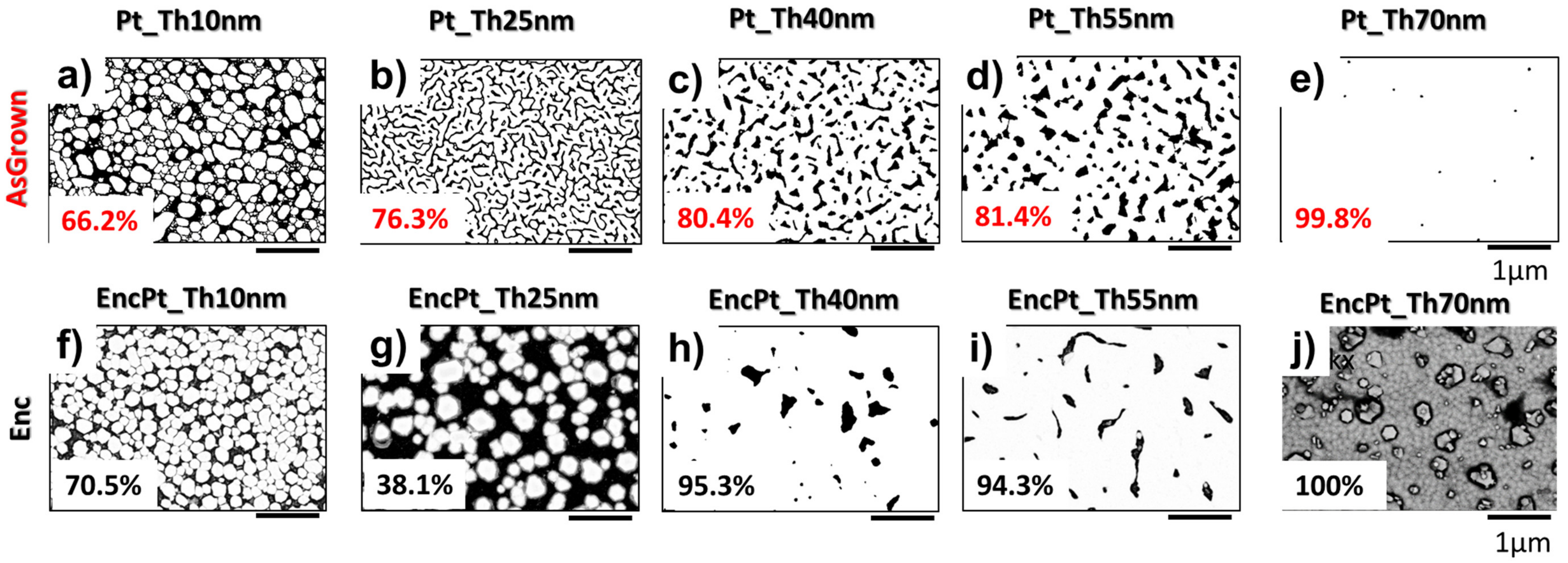

Figure S4: (**a–e**) Variation of the *FWHM_Ang* with the reflection order for Pt films in the as-grown and encapsulated state for the thicknesses Th_{Pt} = 10, 25, 40, 55, and 70 nm. (**f–j**) Variation of the *FWHM_Rad* with the reflection order for as-grown and encapsulated state Pt films with the thicknesses Th_{Pt} = 10, 25, 40, 55, and 70 nm.

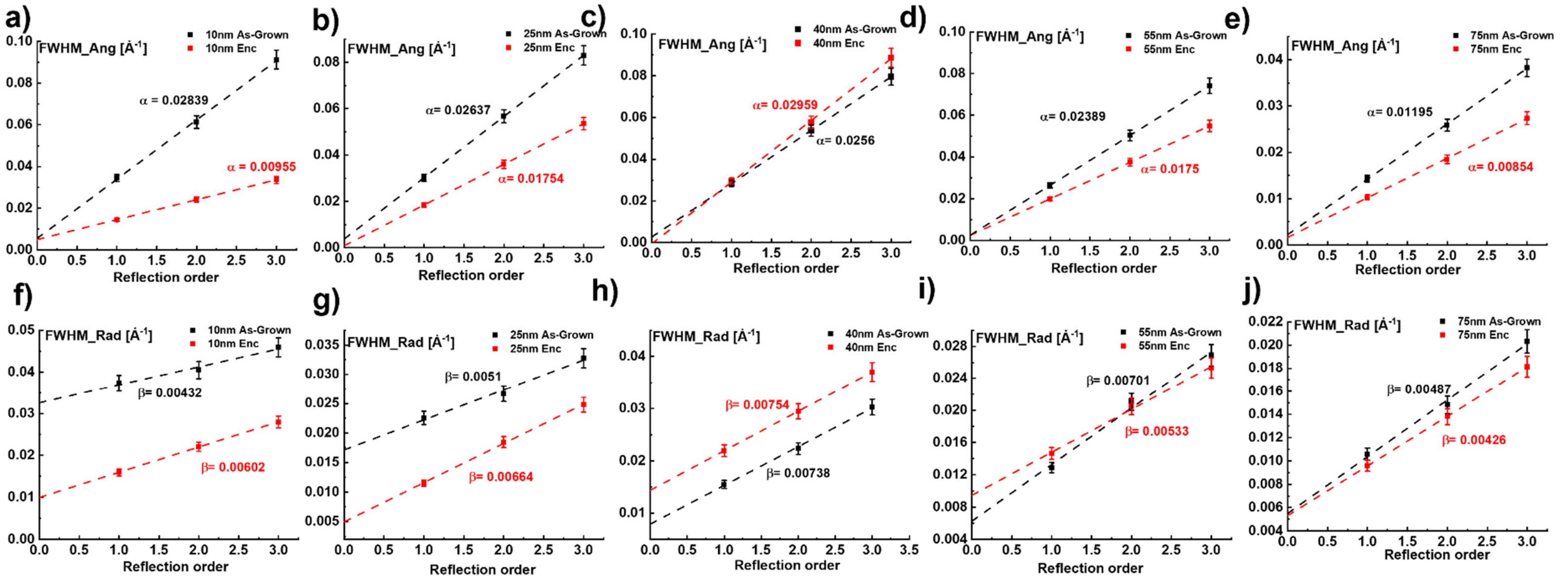

Figure S5. (a) The unit cell of cubic platinum (space group Fm-3m) and (b) a projection of the unit cell along the [111] direction. Different crystallographic planes are indicated by colors: the (111) plane is shown in purple, (-1-10) plane in red, and (11-2) one in blue.

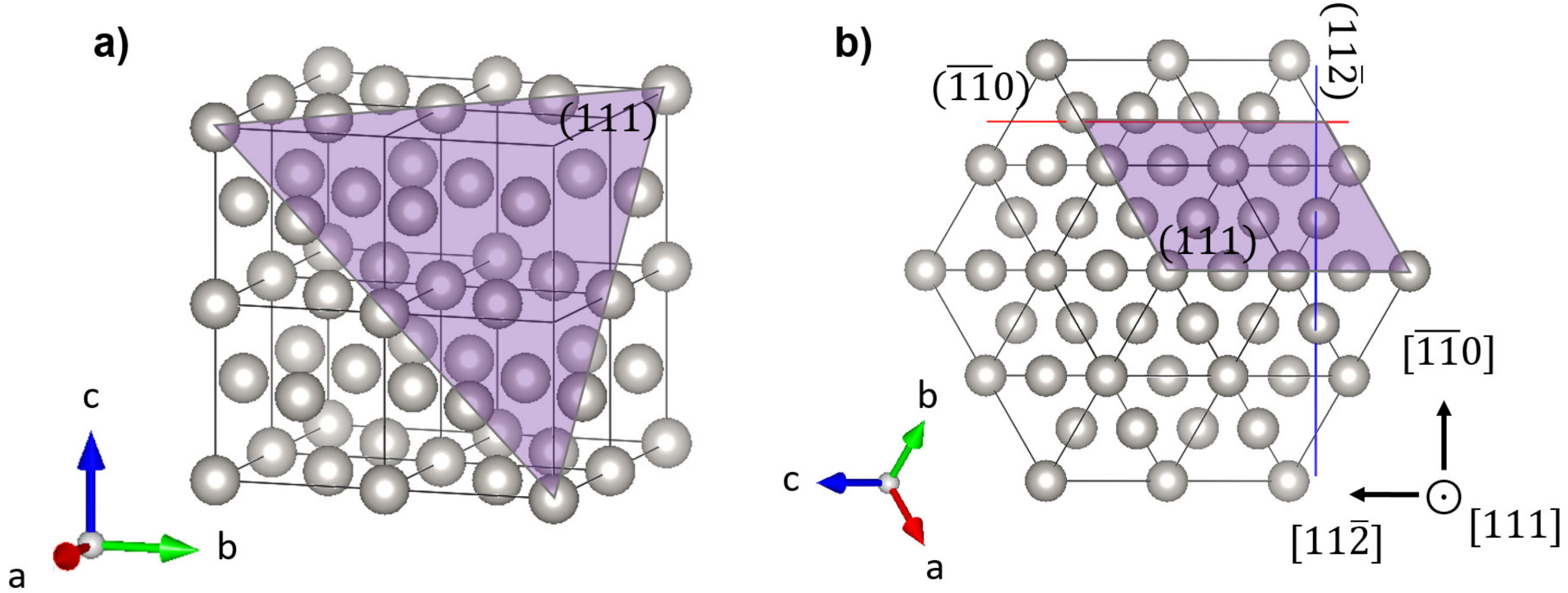

Supplement: Supplementary file 1 [file nanomaterials-14-00725-s001.zip › nanomaterials-2936534-supplementary.pdf]
